# Isolation and Characterization of a Green-Tissue Promoter from Common Wild Rice (*Oryza rufipogon* Griff.)

**DOI:** 10.3390/ijms19072009

**Published:** 2018-07-10

**Authors:** Mande Xue, Yan Long, Zhiqiang Zhao, Gege Huang, Ke Huang, Tianbao Zhang, Ying Jiang, Qianhua Yuan, Xinwu Pei

**Affiliations:** 1 MOA Key Laboratory on Safety Assessment (Molecular) of Agri-GMO, Institute of Biotechnology, Chinese Academy of Agricultural Sciences, Beijing 100081, China; mandexue@gmail.com (M.X.); longyan@caas.cn (Y.L.); Zhangtianbao274200@126.com (T.Z.); 2 College of Tropical Agriculture and Forestry, Hainan University, Haikou 570228, China; zzq19900317@126.com (Z.Z.); Luolengshuang@126.com (G.H.); Huangke199309@163.com (K.H.); 3 Experimental Center Basic Medical Teaching, Capital Medical University, Beijing 100069, China

**Keywords:** common wild rice, Promoter, Green tissue-specific expression, light-induced

## Abstract

Promoters play a very important role in the initiation and regulation of gene transcription. Green-tissue promoter is of great significance to the development of genetically modified crops. Based on RNA-seq data and RT-PCR expression analysis, this study screened a gene, *OrGSE* (GREEN SPECIAL EXPRESS), which is expressed specifically in green tissues. The study also isolated the promoter of the *OrGSE* gene (OrGSEp), and predicted many cis-acting elements, such as the CAAT-Box and TATA-Box, and light-responding elements, including circadian, G-BOX and GT1 CONSENSUS. Histochemical analysis and quantification of GUS activity in transgenic *Arabidopsis thaliana* plants expressing GUS under the control of OrGSEp revealed that this promoter is not only green tissue-specific, but also light-inducible. The ability of a series of 5’-deletion fragments of OrGSEp to drive GUS expression in *Arabidopsis* was also evaluated. We found that the promoter region from −54 to −114 is critical for the promoter function, and the region from −374 to −114 may contain core cis-elements involved in light response. In transgenic rice expressing GUS under the control of OrGSEp, visualization and quantification of GUS activity showed that GUS was preferentially expressed in green tissues and not in endosperm. OrGSEp is a useful regulatory element for breeding pest-resistant crops.

## 1. Introduction

Promoters are key regulators of transcription and also play critical roles in genetic engineering [1]. Promoters can be divided into three types: constitutive, inducible and tissue-specific. Constitutive promoters are widely used in plant genetic engineering [2]. The cauliflower mosaic virus (CaMV) 35S promoter, which drives the expression of genes in almost all tissues, is an important constitutive promoter in dicotyledonous plants [3]. The *Actin*1 promoter from rice is a classical constitutive promoter [4]. However, inducible promoters greatly increase the transcription level of genes under specific physical or chemical signals [5], this is different from constitutive promoters. At present, a great number of inducible promoters have been isolated, including light-inducible, heat-inducible and trauma-inducible [6,7,8,9,10]. Abiotic stress is a major obstacle for crop production, and the promoters of many genes related to abiotic stress have been cloned and applied in crop biotechnology. Five cold-inducible promoters have been isolated from rice, and these promoters can be applied to engineer plants that are resistant to cold stress [11]. The stress-inducible promoter of *TaSnRK*2.8, which is an important gene for wheat response to abiotic stress, has been isolated and characterized, and this promoter can be used to engineer plants with resistance to various abiotic stresses [12].

Because gene expression is driven in specific tissues and development stages, tissue-specific promoters, also called organ- or cell-specific promoters [13], are different from other promoters [14]. These promoters are significant because they avoid potential negative effects of using constitutive promoters, such as metabolic burden [15,16,17,18,19]. Five non-endosperm tissue-expressed promoters have been isolated from rice, and an exogenous *Cry1Ab* gene (*mCry1Ab*) driven by green tissue-specific promoter was expressed in all tissues except for endosperm [20]. Progress has also been made in identifying promoters that drive expression in roots, which is of interest because crops are faced with root-related pests, pathogens and abiotic stresses. The promoters of the soybean *GmPRP1* and *GmPRP2* genes, which are expressed preferentially in roots [21], were isolated and shown to exhibit root-preferential expression [22].The promoter of the serine/threonine kinase gene *ZmSTK2_USP* was isolated and with the use of a GUS reporter system was found to drive pollen-tissue-specific expression [23]. The promoter of *AtGILT* was shown to drive seed coat-specific expression in *Arabidopsis thaliana*, and further studies demonstrated that in canola this promoter drives expression, specifically in the outer integument of the seed coat, and may be useful for improving canola meal [24].

Green tissue-specific promoters have vast potential in transgenic crop breeding [18], particularly in insect-resistant or herbicide-resistant crops [25]. A growing number of transgenic crops with insect resistance gene expression driven by green tissue-specific promoters have been developed, such as cotton, rice, soybean and maize. Transgenic Bt cotton was developed by driving expression of the *B. thuringiensis* endotoxin (*Cry9C*) genes under the control of the *PNZIP* (*Pharbitis nil* leucine zipper) gene promoter, resulting in preferential expression in plant green tissues as well as lower Bt protein accumulation in transgenic cotton seeds [26]. The *rbcS* promoter is another classical green tissue-specific promoter that has been used to drive expression of the *cry2AX1* gene in rice to confer resistance to leaffolders [27]. The 731-bp 5’ flanking sequence of a potato (*Solanum tuberosum*) gene encoding ribulose-1, 5-bisphosphate carboxylase/oxygenase (rubisco) activase (RCA) was characterized, and GUS reporter gene under the control of StRCAp was expressed throughout the green tissue of light-grown transgenic tobacco seedlings. Further analysis revealed that a 220 bp fragment of StRCAp was sufficient for green-tissue-specific and light-inducible expression [7]. Common wild rice (*Oryza rufipogon* Griff.), which is an ancestor of Asian cultivated rice [28], has abundant genetic diversity and is an important germplasm resource for the improvement of cultivated rice [29]. However, the genes specifically expressed in green tissues in common wild rice have not been reported. Previously, we sequenced the transcriptome of common wild rice and identified root-specific and drought-related genes [30]. In this study, we used this dataset to screen for genes with green tissue-specific expression and cloned the promoter sequence of one of these genes, *OrGSE* (*O. rufipogon* GREEN SPECIAL EXPRESS). The full-length *OrGSE* promoter (OrGSEp) and a series of truncated promoters were fused to the GUS reporter gene to identify putative cis-regulatory elements that confer green tissue-specific and light-inducible expression in *Arabidopsis*. We found that in transgenic rice, GUS driven by the OrGSEp promoter was preferentially expressed in green tissues and not expressed in endosperm and root.

## 2. Results

### 2.1. Expression Pattern of OrGSE in Common Wild Rice

RNA-seq data from a previous study [30] were used to screen candidate common wild rice green tissue-expressed genes with higher expression in leaves than in roots (FPKM value in CL >10, FPKM value in CR is <10 and FPKM value in CL 10 times higher than in CR). A total of 1140 unigenes were identified as candidate green tissue-specific expressed genes (Table S3). A series of RT-PCR experiments were performed for confirming the RNA-seq data and combining RNA-seq and RT-PCR results, we selected a novel green tissue-specific expressed gene, comp45689_c0. Blast searches against the rice genome annotation project database (available online: http://rice.plantbiology.msu.edu/) showed that this gene corresponds to *LOC_Os08g02210* and encodes an expressed protein, named OrGSE (*O. rufipogon* GREEN SPECIAL EXPRESS), The BLASTP (Basic Local Alignment Search Tool Protein) analysis revealed that OrGSE shares homology with D27 protein in rice, and prediction of subcellular localization shows that OrGSE is located in chloroplast. Based on RNA-seq FPKM expression values from MSU, *OsGSE* is highly expressed in green tissue; the FPKM value in 20-day-old leaves is >200, but almost 0 in anthers and seeds (Figure S1).

**Figure 1:**
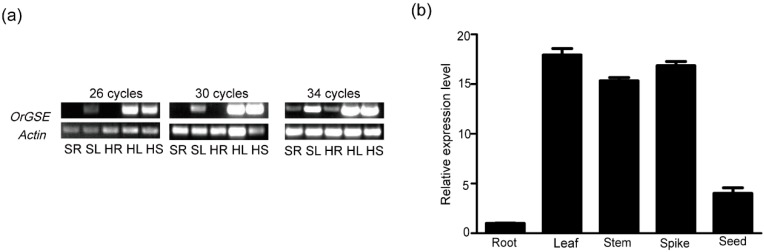
*OrGSE* gene expression in roots and shoots of common wild rice. The mRNA levels of *OrGSE* gene were determined in different tissues and developmental stages by RT-PCR with 26, 30 and 34 cycles of amplification. (**a**) The gel figure of RT-PCR. The rice Actin gene was used as an internal control. SR: Shooting stage Root, SL: Shooting stage Leaf, HR: Heading stage Root, HL: Heading stage Leaf, and HS: Heading stage Stem; (**b**) qPCR analysis of transcript levels of *OrGSE* in different tissues. Data are the means of three replicates, and error bars show the standard error.

RT-PCR and qPCR experiments confirmed that the *OrGSE* gene is expressed in leaves, stems and spikes, but not in roots and seeds (Figure 1). In addition, preferential expression in green tissue was observed at both the seedling and heading stages (Figure 1).

### 2.2. Cloning and Sequence Analysis of the OrGSE Promoter (OrGSEp-374)

**Figure 2:**
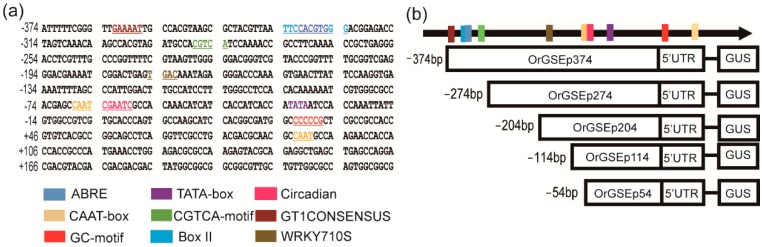
The location of putative cis-acting elements in OrGSEp-374 predicted by the PlantCARE database and schematic diagrams of promoter deletion constructs. (**a**) Putative cis-acting elements in OrGSEp-374, The 5′-region of the *OrGSE* gene containing the 374 bp promoter sequence and 166 bp sequence downstream of the translational start site. The transcription initiation site is defined as +1. The TATA box, CAAT box and other key cis-acting elements are underlined with and indicated by different colors as shown in the legend. The position of each element is also indicated by schematic diagrams; (**b**) The schematic diagrams of the truncated OsGSE-374 constructs. The numbers to the left of these diagrams indicate the position of the 5′-deletion.

A 561 bp fragment including the −374 to +187 region (where the TIS is +1) upstream of *OrGSE* was isolated from common wild rice as the candidate promoter sequence. This promoter fragment, named OrGSEp-374, was submitted to PlantCARE to predict putative cis-elements involved in the regulation of gene expression (Figure 2). Potential regulatory elements were identified within OrGSEp-374 (Table S1), including core elements, such as the TATA-Box, CAAT-Box and GC-Box and cis-elements known to be involved in stress response, such as ABRE (abscisic acid responsive element), CGTCA-motif (response to methyl jasmonate), and LTR (low-temperature response). In addition, a few elements in OrGSEp-374 have been shown to participate in tissue-specific expression, such as the CCGTCC-box (related to meristem-specific activation), the GCN4_motif (endosperm expression) and the Skn-1_motif (required for endosperm expression). Other regulatory elements are involved green tissue-specific expression; most of these elements are light-responsive, such as ACE, G-Box, box II and GT1CONSENSUS. A core element (circadian) involved in circadian control was also identified [31,32].

### 2.3. Spatiotemporal Expression Patterns of OrGSEp-374 and 5’-Deletion Fragments in Arabidopsis

**Figure 3:**
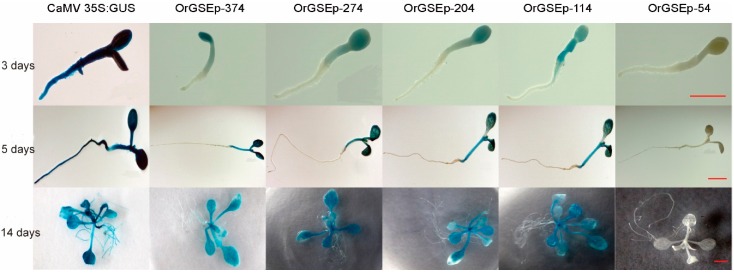
GUS histochemical assays in transgenic *Arabidopsis* T3 seedlings. GUS histochemical assays in transgenic *Arabidopsis* T3 seedlings harboring constructs with GUS expression driven by the CaMV 35S promoter (35S: GUS), OrGSEp-374 (OrGSEp-374) and different 5′-deletion fragments (OrGSEp-274, OrGSEp-204, OrGSEp-114 and OrGSEp-54), during vegetative growth. Photographs were taken 3 days, 5 days and 14 days after seed germination.Bar = 1 cm.

**Figure 4:**
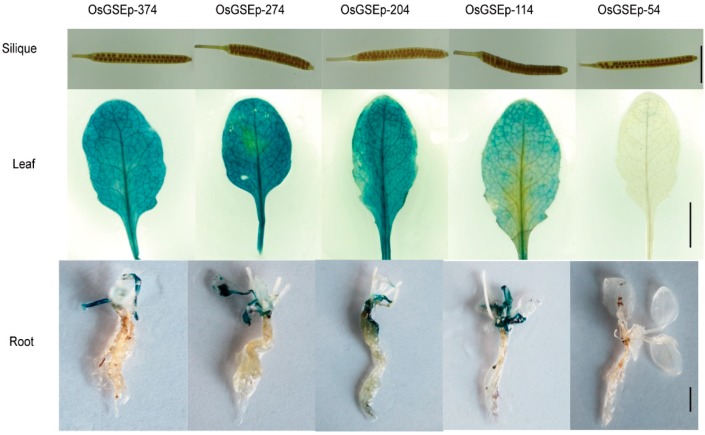
GUS staining in siliques, leaves and roots sampled during the reproductive stage from transgenic T3 *Arabidopsis* seedlings carrying OrGSEp-374, OrGSEp-274, OrGSEp-204, OrGSEp-114 and OrGSEp-54.Bar = 1 cm.

OrGSEp-374 and 5′-deletion reporter constructs were transformed into *Arabidopsis* for promoter functional analysis. OrGSEp-374-driven GUS expression was monitored during different developmental stages and in various organs by histochemical staining. GUS expression was detected in the cotyledons and hypocotyls of 3-day-old seedlings (Figure 3). GUS expression was also detected in the leaves of 5-day- and 14-day-old seedlings, and GUS expression level was higher in leaves than in cotyledons (Figure 3). No GUS expression was observed in roots at any stage (Figure 3). During the reproductive stage, GUS expression was observed in leaves, but not in roots or siliques (Figure 4), indicating that OrGSEp-374 drives expression specifically in green tissues.

**Figure 5:**
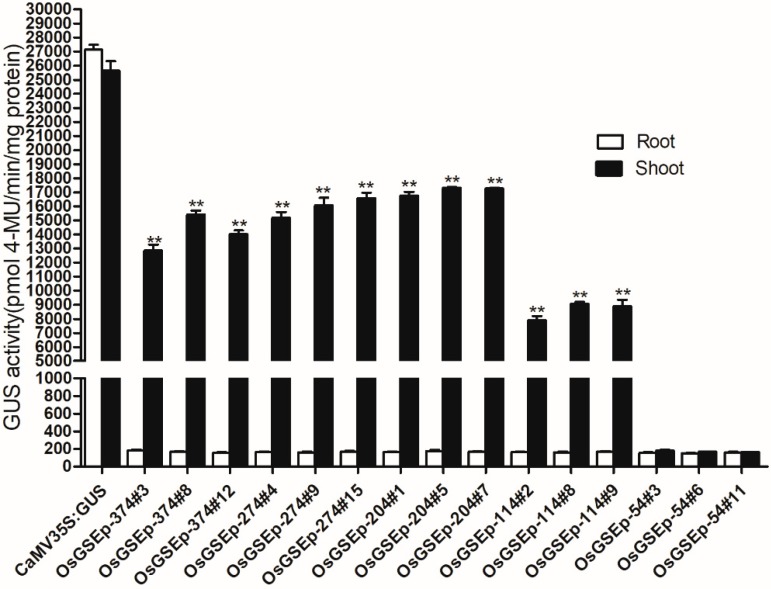
Quantification of GUS activity in transgenic T3 *Arabidopsis* roots and shoots carrying the CaMV 35S promoter, OrGSEp-374 and different 5′-deletion fragments constructs. Gus activity measurements are shown for three positive transgenic lines for each OrGSEp promoter construct. CaMV35S: GUS is an independent transgenic plant carrying the CaMV35S promoter construct. OrGSEp-374#3, OrGSEp-374#8 and OrGSEp-374#12 are three positive transgenic lines carrying the OrGSEp-374 construct. OrGSEp-274#4, OrGSEp-274#9 and OrGSEp-274#15 are three independent transgenic lines carrying the OrGSEp-274 construct. OrGSEp-204#1, OrGSEp-204#5 and OrGSEp-204#7 are three positive lines carrying the OrGSEp-204 construct. OrGSEp-114#2, OrGSEp-114#8 and OrGSEp-114#9 are three lines carrying the OrGSEp-114 construct. OrGSEp-54#3, OrGSEp-54#6 and OrGSEp-54#11 are three transgenic lines carrying the OrGSEp-54 fragment. Data are the means of three replicates, and error bars show the standard error. The “**” indicates that a significant difference (*p* < 0.001) in GUS activity was detected between root and shoots of plants carrying the same promoter construct.

To identify the core elements responsible for green tissue-specific expression, we cloned four different 5′-deletion fragments (Figure 2) into the pBinGlyRed-GUS vector, and transformed these constructs into *Arabidopsis*. Histochemical staining was performed on 3-, 5- and 14-day-old T3 transgenic seedlings. As shown in Figure 3, strong GUS expression was driven by OrGSEp-274, OrGSEp-204 and OrGSEp-114, but no GUS expression was observed in any tissues or stages when GUS was under the control of OrGSEp-54. This result shows that the promoter region from −114 to −54 may contain a key element controlling promoter activity. However, GUS driven by OrGSEp-114 showed weaker expression in leaves than GUS driven by OrGSEp-204, indicating that the promoter region from −204 to −114 may contain an enhancer element (Figure 4). Fluorometric analysis of GUS activity also confirmed that GUS expression driven by OrGSEp-204 was higher than when driven by OrGSEp-114. The results of fluorometric GUS assays were consistent with histochemical staining (Figure 5), supporting the promoter fragment from −54 to −114 playing a critical role in the promoter activity.

### 2.4. OrGSEp-374 Confers Light-Responsive Expression

**Figure 6:**
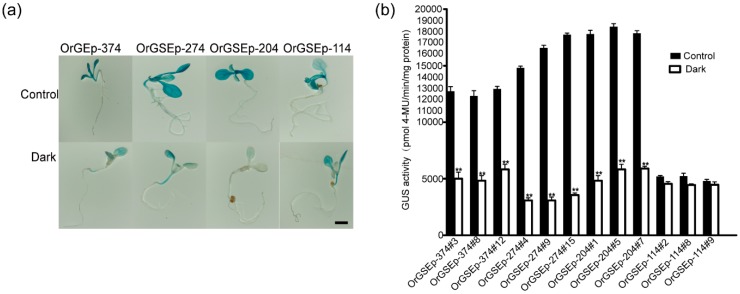
GUS staining and quantification of GUS activity of transgenic *Arabidopsis* seedlings containing different 5’-promoter deletion fragments grown under light and dark conditions. (**a**) Ten-day-old seedlings were stained to observe GUS expression. Bar = 1 cm; (**b**) Quantification of GUS activity in 20-day-old T3 transgenic *Arabidopsis* grown under light and dark conditions. OrGSEp-374#3, OrGSEp-374#8 and OrGSEp-374#12 are three positive transgenic lines carrying the OrGSEp-374 construct. OrGSEp-274#4, OrGSEp-274#9 and OrGSEp-274#15 are three independent transgenic lines carrying the OrGSEp-274 construct. OrGSEp-204#1, OrGSEp-204#5 and OrGSEp-204#7 are three positive lines carrying the OrGSEp-204 construct. OrGSEp-114#2, OrGSEp-114#8 and OrGSEp-114#9 are three lines carrying the OrGSEp-114 construct. Data are the means of three replicates, and standard errors are shown by error bars. The “**” indicates that a significant difference (*p* < 0.001) in GUS activity was detected between roots and shoots of seedlings carrying the same promoter construct.

OrGSEp-374 sequence analysis showed that the promoter contained many cis-acting elements involved in light responsiveness, such as ACE, Box II, and GT1CONSENSUS (Figure 2). We performed GUS staining and fluorometric assays to determine whether the expression of *OrGSE* was regulated by light. GUS staining showed that *OrGSE* expression was induced by light (Figure 6), and quantitative fluorometric analysis confirmed this result (Figure 6). To find the core elements related to light responsiveness, we analyzed the light-inducible activities of 5′-deletion fragments of OrGSEp-374. GUS expression driven by OrGSEp-374, OrGSEp-274 and OrGSEp-204 was similar under light conditions; however, GUS activity driven by these promoters was significantly reduced in the dark. Interestingly, the pattern of GUS expression in OrGSEp-114-GUS lines was the same under both light and dark conditions (Figure 6). These results demonstrate that the promoter fragment from −374 to −114 contains vital elements involved in light response, but the promoter fragment from −114 to +1 does not. This is consistent with the presence of elements involved in light response in the OrGSEp-374 predicted by PlantCARE (Figure 2; Table S1).

### 2.5. The Expression Pattern of OrGSEp-374 in Rice

**Figure 7:**
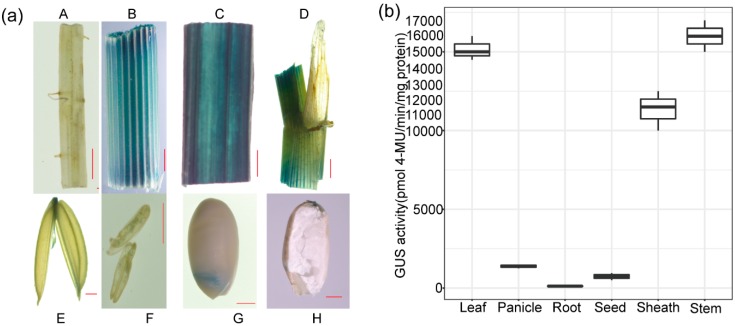
GUS histochemical assays and GUS activity in different tissues of OrGSEp-374 transgenic rice. (**a**) GUS histochemical assays of OrGSEp-374 transgenic rice. A. Root, B. stem, C. leaf, D. ligule, E. spikelet, F. anther, G. seed. H. endosperm. Bar = 1000 μm; (**b**) GUS activity in different tissues of transgenic rice carrying the OrGSEp-374 construct. Boxplots show GUS activity in different tissues of transgenic rice. The lower boundary of each box denotes the 25th percentile, the upper boundary of each box denotes the 75th percentile, and the solid line in the middle of each box denotes the 50th percentile. The two ends of the error bars denote the maximum and minimum values.

GUS staining and fluorometric quantification was performed on four independent lines of rice containing the OrGSEp-374 construct. Strong GUS expression was observed in the stem and leaf, weak expression was observed in the anther, ligule, spikelet and embryo, and no expression was observed in the root and endosperm (Figure 7). Further fluorometric analysis of GUS activity showed that the average specific activities in transgenic rice leaves and stems exceeded 15,000 pmol 4-MU min^−1^ mg^−1^ total proteins. However, the GUS activity in the root and panicle was less than 1000 pmol 4-MU min^−1^ mg^−1^ total proteins, which was far lower than the activity level in leaves and stems (Figure 7). GUS activity in the seed was slightly higher than in roots because GUS was weakly expressed in the embryo (Figure 7).

## 3. Discussion

Next-generation sequencing has become a new method for exploring transcriptional regulation and has been used to identify cis-elements involved in gene regulation. To date, many important genes and regulatory elements have been isolated by next-generation sequencing. Using RNA-seq analysis, more than 87 transcription factor genes were identified as being expressed during seed development and fatty acid accumulation. One of these genes, GmDREBL, which belongs to the DREB subfamily of the AP2 family, was shown to participate in fatty acid accumulation based on analysis of *Arabidopsis* plants transformed with the *GmDREBL* gene driven by 35S promoter [33]. These studies illustrate that high-throughput sequencing can be used to identify genes and regulatory elements. In this study, we identified 1140 candidate genes with preferential expression in green tissues by screening an RNA-seq library [30] and performing traditional RT-PCR. We screened a green tissue-specific gene, *OrGSE*, and the promoter of this gene was isolated. GUS reporter gene driven by the promoter was strongly expressed in leaves and stems.

OrGSE shares homology with D27 protein, which is located in chloroplasts. D27 is involved in MAX/RMS/D pathway, in which D27 as a member participates in the biosynthesis of strigolactones, regulating rice tiller bud outgrowth [34]. Although the function of *OrGSE* is unknown, the sequence of *OrGSE* is highly similar with D27. *OrGSE* may share similar function with D27. In *Arabidopsis*, AtD27 is required for the inhibition of secondary bud outgrowth by Strigolactones [35]. According to the sequence similarity between OrGSE and D27, we can infer OrGSE may locate in chloroplasts and participate in rice tiller outgrowth, which can account for OrGSEp drive gene expressed in green tissues.

The tissue-specific expression of transgenes plays a critical role in biotechnology crops because it avoids fitness costs caused by constitutive expression of target genes [9]. However, the information about the core cis-acting elements controlling tissue-specific expression is limited. Two novel cis-elements controlling tissue-specific expression, namely PSE1 (panicle/stem-specific element 1) and LPSRE2 (leaf, panicle/stem and root element 2), were identified in the *DX1* promoter by deletion analysis and gel mobility shift assays [35]. Five tissue-specific expression-related cis-elements in the green tissue-specific promoter PD540 were also characterized. These elements include GEAT, WRKY71OS and TGAC, and are used as references for discovering novel tissue-specific promoters. In this study, we showed that OrGSEp-114 is a short green tissue-specific promoter. However, the above described cis-elements were not found in this novel promoter. The OrGSEp-114 promoter may contain new cis-elements involved in green tissue-specific expression. Our future research will focus on screening cis-acting elements controlling green tissue-specific expression in this promoter by electrophoretic mobility shift assay (EMSA), including at least one novel green tissue-specific element. The OrGSEp-114 has promoter activity and green-specific distinguishing feature, but OrGSEp-54 cannot drive reporter gene expression, which means that 114 bp region of this promoter was sufficient to drive green tissue-specific expression. Cis-elements show that the promoter contains one CAAT-box and GC-motif from −114 to −54. This CAAT-box and GC-motif may be vital to promoter activity (Table S1). Moreover, the promoter from −114 to −54 contains one circadian cis-element, and many studies show that circadian elements participate in green tissue-specific expression. That information will support OrGSEp-114 being a short green tissue-specific promoter.

Light is essential for photosynthesis, and many green tissue-specific promoters contain light-inducible elements [36]. The *IbRbcS* gene from sweet potato shows green tissue-preferential expression and the *IbRbcS1* promoter confers light-responsive expression in transgenic *Arabidopsis* [37]. In this study, results showed that OrGSEp-374, as with the *IbRbcS1* promoter, has both green tissue-specific and light-inducible expression. Based on analysis of the activity of the full-length and truncated promoters in transgenic *Arabidopsis*, we showed that the OrGSEp-374 promoter contains cis-elements involved in light responsiveness, such as ACE, G-box and GT1CONSENSUS, which may regulate green tissue-specific and light-induced expression. Furthermore, analysis of 5′-deletions of this promoter showed that OrGSEp-274 and OrGSEp-204 also have light-induced activity, so the promoter may contain more than one core light-inducible element.

Many insect and fungal diseases influence the normal growth of rice and cause severe yield loss. Sheath blight-resistant rice has been developed by co-expressing the *chitinase* and *oxalate oxidase 4* genes in green tissues under the control of the green-specific *rbcS* promoter in rice [38]. A transgenic potato resistant to potato tuber moths with 100% tuber moth larval mortality was developed by expressing cry1Ab under the control of the *PEPC* promoter, a green tissue-specific and light-inducible promoter from maize [39]. Cry9C driven by *PNZIP* promoter, a green tissue promoter from *Pharbitis nil*, was used to develop transgenic pest-resistant cotton, and the accumulation of cry9C protein in seeds was 100 times lower than that observed for the seeds of the CaMV 35S:Cry9C line [40]. In our study, we have isolated a novel rice green tissue-specific promoter that does not drive expression in endosperm, and the GUS activity in seeds was much lower than in green tissues. Although the gene driven by this promoter is slightly expressed in embryos, the embryo is linked to bran and will be discarded with the bran during rice bran desquamation, thus alleviating concerns about food safety. OrGSEp is not only not expressed in endosperm but is also not expressed in roots. Thus, the use of this promoter in transgenic crops will ease food safety concerns and reduce the waste of resources that occurs with constitutive expression. This promoter provides a new element for developing insect-resistant and disease-resistant rice and other crops.

## 4. Materials and Methods

### 4.1. Plant Materials and Growth Conditions

Common wild rice seeds were collected from Guangdong Province in China. The collection was approved by the supervision department of Guangdong wild rice protection. Plants were grown in a greenhouse under a 12 h light/12 h dark cycle at 28 °C. *Arabidopsis thaliana* (Col-0) seeds were surface-sterilized with 75% (*v*/*v*) ethanol for 10 min and washed for 1 min with 95% (*v*/*v*) ethanol. The sterilized *Arabidopsis* seeds were spread on plates containing 1/2 Murashige and Skoog medium. After stratification at 4 °C for 2 days, the plates were transferred to a plant growth incubator, and seeds were germinated under a 16 h light/8 h dark cycle at 22 °C–24 °C.

### 4.2. Screening of Green Tissue-Specific Genes and Expression Analysis

Based on RNA-seq data from our previous study [30], genes with higher FPKM (Fragments Per Kilobase per Million) in CL (Control Leaf) than in CR (Control Root) were chosen as candidate green tissue-specific promoter-regulated genes. RT-PCR and Real-time quantitative PCR (qPCR) were used to confirm gene expression patterns. According to expression data, a candidate gene (*OrGSE*) was selected for subsequent analysis. The upstream sequence of *OrGSE* was regarded as a candidate green tissue-specific promoter.

Single-stranded cDNA was synthesized from the total RNA isolated from common wild rice leaves using the 5X All-In-One RT Master Mix (Applied Biological Materials, Vancouver, VAN, Canada). The sequence of *OrGSE* was obtained from a cDNA library. We analyzed sequence conservation by performing a BLAST search against the Michigan State University Rice Genome Annotation Project Database (MSU). We designed a pair of primers (OrGSE-F and OrGSE-R) to amplify the *OrGSE* sequence from leaf cDNA. The PCR amplification with 2× Phanta™ Master Mix was performed according to the manufacturer’s instructions (Vazyme, Nanjing, China). The PCR products were purified, cloned into the pEASY-Blunt Cloning Vector (Transgene, Beijing, China) and sequenced. The expression level of *OrGSE* in common wild rice roots, stems and leaves was analyzed using RT-PCR and qPCR. *Actin1(LOC_Os03g61970)* was used as an internal control. Different numbers of cycles were used in RT-PCR amplification for *OrGSE* expression analysis. qRT-PCR was performed using the gene-specific primers listed in Supplemental Table S2 (Actin-F/R and OrGSE-F/R) and a real-time PCR7500 system (Applied Biosystems). Data were collected using the ABI PRISM 7500 sequence detection system. Three biological replicates with independent mRNA isolations were performed, each with three technical repeats, the rice Actin gene was used as an internal control, and the mRNA relative expression level was calculated using the 2^−ΔΔ*C*T^ method.

### 4.3. Cloning of the OrGSE Promoter and Sequence Analysis

Genomic DNA was isolated from common wild rice leaves using the EasyPure Plant Genomic DNA Kit (Transgene Biotech, Beijing, China) and used as the template for amplification of the *OrGSE* promoter. The PCR products were purified and cloned into the plant expression vector pCAMBIA1305.1 using the In-fusion method. This construct was used for sequencing and promoter activity analysis.

Core regulatory elements in the promoter sequences were predicted using the online tool plantCARE (available online: http://bioformatics.psb.ugent.be/webtools/plantcare/html/) [41].

### 4.4. PCR Amplification of 5’-Deletion Fragments of the OrGSE Promoter

We designed five forward primers with *Bam*H I restriction sites (F-374, F-274, F-204, F-114, F-54) and one reverse primer with an *Eco*R I restriction site (R) to obtain 5′-deletion fragments of the OrGSE promoter. These primers were designed to amplify regions −374 (F-374/R), −274 (F-274/R), −204 (F-204/R), −114 (F-114/R), and −54 (F-54/R) upstream of the transcription initiation site (TIS), which was designed as +1. The PCR cycling parameters were as follows: 95 °C for 3 min, 35 cycles of 95 °C for 15 s, 60 °C for 15 s, and 72 °C for 1 min followed by 72 °C for 5 min. The full-length promoter and 5’-deletion fragments were cloned into a modified pBinGlyRed vector, which includes GUS plus-his6 that was inserted into the multiple cloning site using the *Eco*R I and *Xma* I restriction sites (Figure S2). The five promoter fragments were named OrGSEp-374, OrGSEp-274, OrGSEp-204, OrGSEp-114 and OrGSEp-54. The constructs were then introduced into *Arabidopsis thaliana* (Col-0), *Agrobacterium*-mediated transformation of *Arabidopsis* was performed through floral dipping [42]. We also amplificatedOrGSEp-374 fragment by 1305GSEp-F/R primers, and it was inserted into pCAMBIA1305.1 vector by *Hin*d III and *Nco* I restriction sites, the construct was introduced into Nipponbare (*Oryza sativa* L. ssp. *japonica*) by *Agrobacterium*-mediated transformation.

### 4.5. Detection of the Expression Pattern of the OrGSEp Promoter and 5’-Deletion Fragments in Different Organs

For GUS histochemical assays, homozygous T3 transgenic *Arabidopsis* seedlings (3-, 5- and 14-day-old) were incubated in GUS staining solution overnight. During the reproductive stage, GUS histochemical assays were performed for transgenic *Arabidopsis* leaves, siliques and root. GUS activity was quantified in the leaves and roots of 3-week-old seedlings. Three independent lines were selected for each promoter deletion construct.

### 4.6. Inducible Activity Analysis of the OrGSEp Promoter and 5’-Deletion Fragments

To analyze the light inducible activity of the promoter and different 5′-deletion fragments, 3-week-old transgenic *Arabidopsis* plants were grown in the dark for 24 h. Then, the leaves were frozen in liquid nitrogen and stored at −80 °C for GUS fluorometric assay. The control plants were placed under natural growth conditions. For GUS staining, 10-day-old seedlings were placed in the dark for 24 h and transferred to natural condition for 24 h, and control plants were kept under natural conditions.

### 4.7. GUS Histochemical and Fluorometric Analysis

GUS histochemical and fluorometric analysis was performed as previously described (Wu et al., 2003). Transgenic *Arabidopsis* seedlings at different growth stages and various tissues at the reproductive stage (as described above) were incubated in GUS staining solution (Coolaber, Beijing, China) at 37 °C overnight, and then the samples were cleared with 75% (*v*/*v*) ethanol. GUS staining was observed under a ZEISS Stemi 508 microscope and photographed with a SONY camera.

Protein was extracted from 100 mg root and leaf tissue from transgenic *Arabidopsis* plants. The protein concentrations were determined using the Bradford method with bovine serum albumin (BSA) as the standard. GUS activity was determined fluorometrically by measuring the amount of 4-methylumbelliferone (4-MU) produced by GUS per milligram of total protein per minute [43]. GUS activity was measured for three lines for each promoter construct, and three replicates were performed for each line. The error bars are reported as ‘‘mean ± standard error’’. The boxplot was drawn by R language gglpot2 package.

## Supplementary Materials

Supplementary materials can be found at http://www.mdpi.com/1422-0067/19/7/2009/s1.
