# Supplementary material for: Isolation and Characterization of a Green-Tissue Promoter from Common Wild Rice (Oryza rufipogon Griff.)
Source: Int J Mol Sci. 2018 Jul 10;19(7):2009. doi: 10.3390/ijms19072009 (PMC6073244; doi:10.3390/ijms19072009)
Supplement: Supplementary file 1 [file ijms-19-02009-s001.zip › ijms-320659-Supplementary materials/Figure S1.docx]

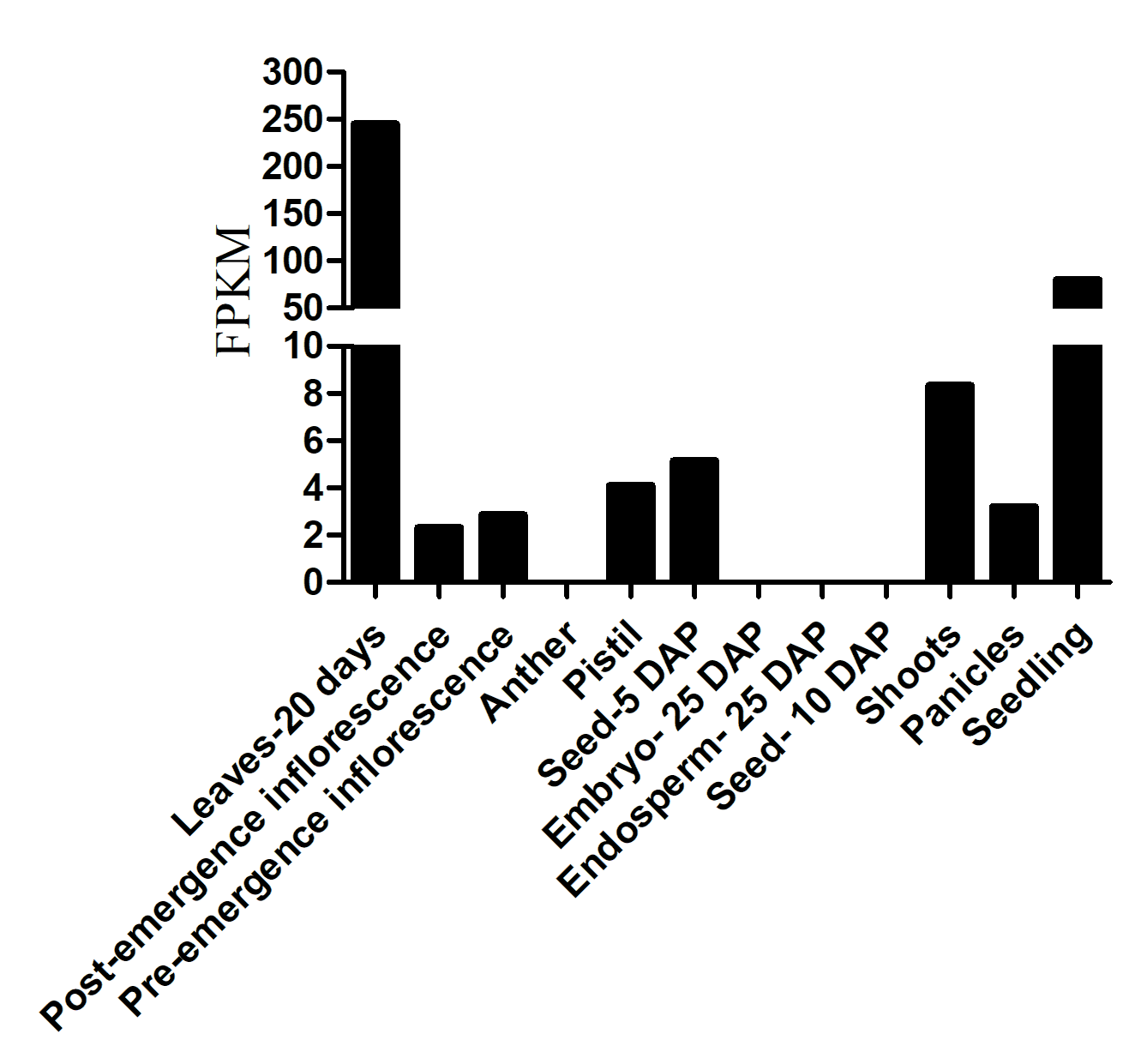


**Figure S1** RNA-Seq FPKM Expression Values of OsGSE in different tissue in different stages

The data was obtained from MUS(<http://rice.plantbiology.msu.edu/>)
