# Supplementary material for: Isolation and Characterization of a Green-Tissue Promoter from Common Wild Rice (Oryza rufipogon Griff.)
Source: Int J Mol Sci. 2018 Jul 10;19(7):2009. doi: 10.3390/ijms19072009 (PMC6073244; doi:10.3390/ijms19072009)
Supplement: Supplementary file 1 [file ijms-19-02009-s001.zip › ijms-320659-Supplementary materials/Table S1.docx]

**Table S1 Putative cis-acting elements in the OrGSEp-374 by PlantCARE**

| Name of cis-element | Sequence | Position from transcription start site | Function |
| --- | --- | --- | --- |
| A-box | CCGTCC | -303,-327, | *cis*-acting regulatory element |
| ABRE | CACGTG | -330 | *cis*-acting element involved in the abscisic acid responsiveness |
| ACE | ACGTGGA | -332 | *cis*-acting element involved in light responsiveness |
| CAAT-box | CAAT | -17,-68,-122,-359 | common *cis*-acting element in promoter and enhancer regions |
| CCGTCC-box | CCGTCC | -223,-324 | *cis*-acting regulatory element related to meristem specific activation |
| CGTCA-motif | CGTCA | -287 | *cis*-acting regulatory element involved in the MeJA-responsiveness |
| G-Box | CACGTR | -300,-330,-352 | *cis*-acting regulatory element involved in light responsiveness |
| G-box | CACGTV | -289,-300, -330,-352 | *cis*-acting regulatory element involved in light responsiveness |
| GCN4_motif | CAAGCCA | -305 | *cis*-regulatory element involved in endosperm expression |
| LTR | CCGAAA | -370 | *cis*-acting element involved in low-temperature responsiveness |
| O2-site | GATGACATGG | -35.-290 | *cis*-acting regulatory element involved in zein metabolism regulation |
| Skn-1_motif | GTCAT | -286 | *cis*-acting regulatory element required for endosperm expression |
| TATA-box | TATA | -32 | core promoter element around -30 of transcription start |
| box II | TCCACGTGGC | -332 | part of a light responsive element |
| circadian | CAANNNNATC | -67 | *cis*-acting regulatory element involved in circadian control |
| GT1CONSENSUS | GRWAAW | -362 | cis-acting element involved in light responsiveness |
| GC-motif | GCGC | -79 | common cis-acting element in promoter |
| WRKY710S | TGAC | -175 | repressor element |

R indicates A or G; V indicates A, C or G
