# Supplementary material for: Isolation and Characterization of a Green-Tissue Promoter from Common Wild Rice (Oryza rufipogon Griff.)
Source: Int J Mol Sci. 2018 Jul 10;19(7):2009. doi: 10.3390/ijms19072009 (PMC6073244; doi:10.3390/ijms19072009)
Supplement: Supplementary file 1 [file ijms-19-02009-s001.zip › ijms-320659-Supplementary materials/Table S2.docx]

**Table S2. The primers used in this study**

| Primers | Primer sequence (5’ to 3’) | Fragment length | Function |
| --- | --- | --- | --- |
| F-374 | ACGCGTAAGGGGATCCAGTCGTCGTCGTCGTACGTC | 374bp | Vector constrction |
| F-274 | ACGCGTAAGGGGATCCATCCAAAACCGCCTTCAAAACC | 274bp | Vector constrction |
| F-204 | ACGCGTAAGGGGATCCACCCGGTTTTGCGGTCGAGGGA | 204bp | Vector constrction |
| F-114 | ACGCGTAAGGGGATCCCATTGGACTTGCCATCCTTTGG | 114bp | Vector constrction |
| F-54 | ACGCGTAAGGGGATCCGAATCGCCACAAACATCATCAC | 54bp | Vector constrction |
| R | GATCTACCATGAATTCAGTCGTCGTCGTCGTACGTC |  | Vector constrction |
| 1305GSEp-F | GCAGGCATGCAAGCTTATTTTTCGGGTTGAAAATTGCC | 374bp | Vector constrction |
| 1305GSEp-R | CTCAGATCTACCATGGAGTCGTCGTCGTCGTACGTC |  | Vector constrction |
| Red-F | AGTCGTCGTCGTCGTACGTC |  | Sequencing |
| Red-R | AGTCGTCGTCGTCGTACGTC |  | Sequencing |
| Actin-F | TGCTGGTCGTGATCTGACTG | 239bp | Internal control |
| Actin-R | CTTCTGGGCACCTGAACCTC |  | Internal control |
| OrGSE-F | GTGGGAGTGGGACTACGAGA | 230bp | RT-PCR / qRT-PCR |
| OrGSE-R | ACCTCCACAACCTCTGATGG |  | RT-PCR / qRT-PCR |
