# Supplementary figures and images for: Isolation and Characterization of a Green-Tissue Promoter from Common Wild Rice (Oryza rufipogon Griff.)
Source: Int J Mol Sci. 2018 Jul 10;19(7):2009. doi: 10.3390/ijms19072009 (PMC6073244; doi:10.3390/ijms19072009)

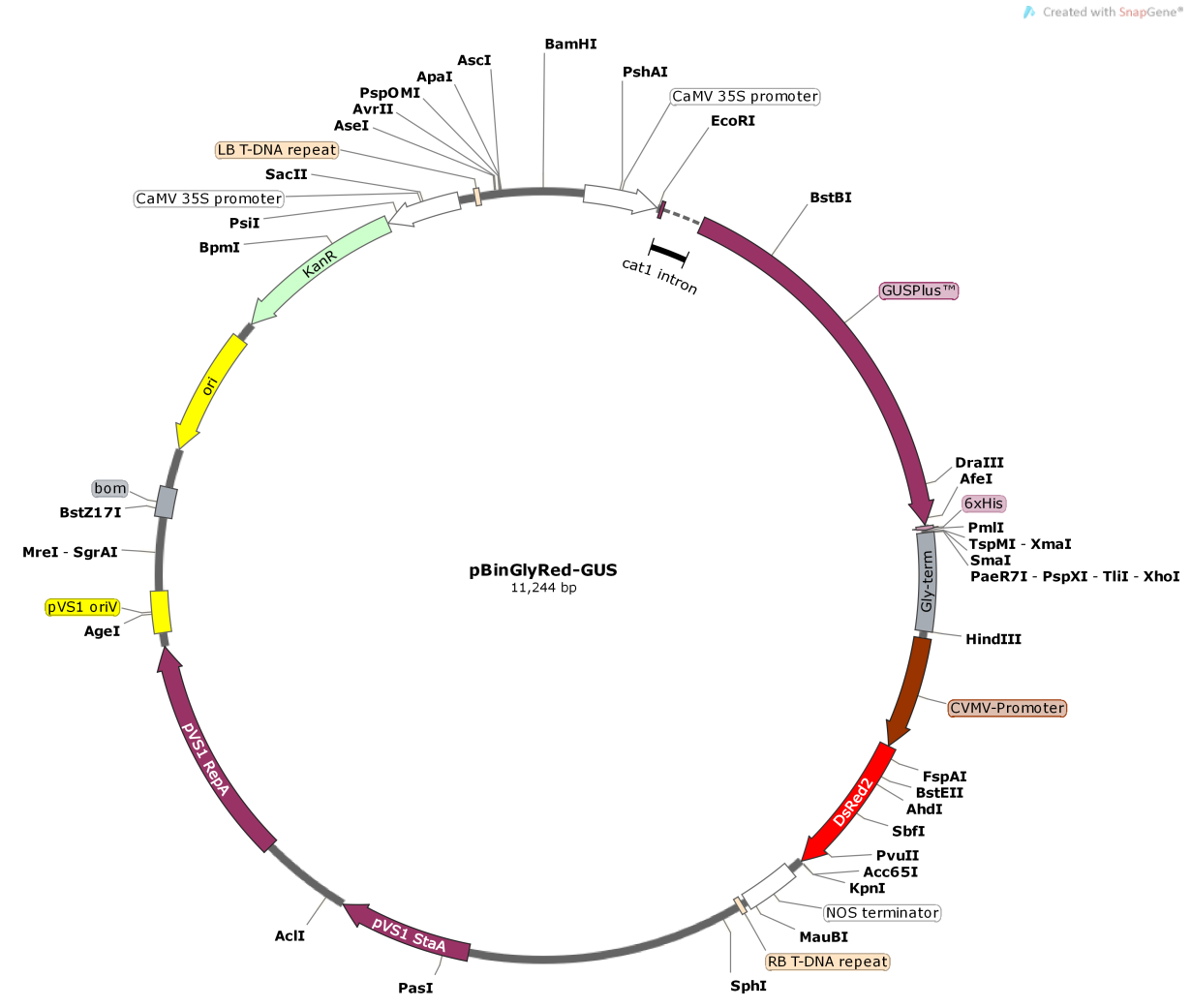


**Figure S2** The pBinGlyRed-GUS vector

Supplement: Supplementary file 1 [file ijms-19-02009-s001.zip › ijms-320659-Supplementary materials/Figure S2.docx]
